# A Comment on Our Professional Status

**Published:** 1905-02-15

**Authors:** H. C. Sexton


### A COMMENT ON OUR PROFESSIONAL STATUS.

BY H. C. SEXTON, D.D.S.

Extract from the Proceedings of the Indiana State Dental Society as published in the February "Summary."

We have our faults, but in this respect we are no worse than other professions. Indeed, I am firmly of the opinion that what the dentist needs to-day is to be a better business man, not a poorer one. It is a mistake for a professional man to get upon stilts and look down upon commercialism. We are all, from the highest professional man to the poorest man, working for self-advancement, and we must not try to deceive ourselves into thinking that we are or should be philanthropists. We are no more moved by greed than are physicians or lawyers.

Dr. Weirick says, "I am tired of seeing the section of stomatology of the American Medical Association lagging perpetually at the rear end of the profession; I want to see an end to the condescension and the patronizing that are always extended to practitioners of our profession by the members of the older branches of medicine—an end to the humiliating position we occupy in the meetings of the American Medical Association."

Now, in the beginning, I led you to believe there was scarcely a statement of the Doctor's that I could agree with, but here I shall have to retract. I, too, am tired of this humiliating position into which we, as a profession, have been thrown. I am as tired, indeed, I suspect, more tired, than he of the condescension and patronizing to which we are subjected. But though we agree in our tiredness, I fear we shall disagree in the methods of cure we advocate.

Let us stand upon our own feet and not be foreverlastingly whining around the medical profession for admission to their ranks, like a sick puppy wanting to be taken in out of the cold. Of late years there has been entirely too much of this boot-licking. Our profession, in our work, is much nearer perfection than is the medical profession in theirs. Then why, in the name of the great two-headed Janus, should we come down from our high estate to seek an inferior station under them? We have been patronized and humiliated, you say. Exactly so, and why? Because by our grovelling we have earned only the contempt of the older profession. We are not wanted in the medical profession. Then why lower ourselves by trying to butt in? Let us quit this sick puppy whimpering, this self-degradation, this boot-licking about men who in their world's work are far, far behind us. We are an independent profession and we are not a specialty in the practice of medicine. Then, like our forefathers of '76, let us sink or swim, live or die, survive or perish on our foundation of freedom of action, freedom of conscience and freedom of name.

In my opinion, the dentist who considers the word "dentist" not good enough for him is a—, a—, well, it would not be polite for me to say just what he is. There is too much twiddle-dum and twiddle-dee, poppycock and fol-de-rol in the makeup of that word stomatologist. To my mind, it is no more an evidence of progress than a wig is of hair. I am not only suspicious but I am certain there is a bald spot underneath.

The essayist says we have undoubtedly done more to ameliorate suffering than any equal number of men in the world. This is true, and furthermore, let me say to you right here that our relief of suffering has been largely through mechanical means and not through medical. Mechanics is our foundation, our indispensable foundation; medicine to us is a side issue, important, but still an incident.

And further the essayist says: "While our representatives who have gone abroad are accorded high places as operators, they are scoffed at as scientists." Here the doctor comes near drawing out the thread of his verbosity finer than the staple of his argument. In other words, our European brothers are better physiologists, pathologists, bacteriologists and microscopists than we. But we can fill teeth and save teeth better than they. Then let the so-called scientists go on studying their wiggle worms, while we go on perfecting ourselves as operators. I, for one, shall have no fear of the result.

We need substances to take the place of unsightly gold; we need light on the best methods of porcelain inlays; we need better mechanical appliances. Does any one, for a moment, think we can obtain information on these points from the medical profession? There is neither rhyme nor reason in this. Many of us make the mistake of thinking that scientific investigation is monopolized by our medical brethren. Is a thing not scientific because mechanical? Science is no more medical, no more chemical, than it is mechanical. Why, mechanics is the very essence of science! In mechanics there is nothing to be ashamed of. It is a definite and a practical science of mathematical accuracy.

We are mechanics; we belong to the highest rank of mechanics; we are members of a mechanical profession. Then let us be content, aye, let us be proud of it, and let us not attempt to put gaudy butterfly wings on youthful busy bees. Let us thank God that we are what we are, and that we are not a puny side-show to the medical experimental circus, as are our brethren in Europe; that we have not descended from our high position of usefulness into the maze of uncertainties and guess-work of medicine, Let us tell the American Medical Association that so far as we are concerned they may abolish their section on stomatology, that we shall remain dentists and shall conduct our scientific investigations without extraneous aid; that our attitude of submission, of inferiority, has been abandoned; that we stand up before all the world an independent profession with entangling and humiliating alliances with none.

Dear old Shakespeare has said:

> "To gild refined gold, to paint the lily,
> To throw a perfume on the violet,
> To smooth the ice or add another hue
> Unto the rainbow, or with taper light
> To seek the beauteous eye of heaven to garnish
> Is wasteful and ridiculous excess."

I think the essayist has scared up a mare's nest in the condition he has pictured of our indebtedness, our bondage, to supply men. His statements come as a great surprise to me. I know of no young dentist over head and ears in debt to supply men, whose equipment is mortgaged, whose very independence is crushed out of him. But, on the other other hand, I know many a young dentist who, through the kindness of a supply man, has been enabled to begin practice and has succeeded. Of course, this was not all kindness on the part of the supply man. It was business, and good business as well. The supply men are not angels. I know several intimately; they are good fellows but without a sign of wings. After all, they are just as the good God made them, only some of them are worse, much as you and I. But even the devil must be given his dues. I have never heard of supply men establishing dental offices as breweries establish saloons, having practical ownership over them and regarding them solely as distributing stations for their goods. I am sure no such condition exists in Indiana.

Now, as to our dental periodicals being all supply-house organs, I must say that that is very nearly correct. We have one notable except in the *International Dental Journal.* We have had other independent journals. They didn't succeed because there is no money in publishing a dental magazine. We must accept the conditions as they are and abide by them. Manufacturers will spend more money in making a dental journal good than will professional men. It pays them to do so. It doesn't pay the other man, so there is an end of the matter. But no one can say the columns of these house organs are not open to all communications. Even Dr. Weirick's paper—quite a severe arraignment of the supply men—will be published by some "house organ" and without a word of alteration. If this doesn't constitute independent journalism it comes very near it. The supply men come to our conventions upon our invitation and I must say their exhibits are often most interesting and instructive. A dental meeting with no exhibits around would be, at least to a country dentist like myself, a very tame affair.

But on following out Doctor Weirick's suggestion that the dental students should be warned and protected from the danger of becoming a slave to the supply man, I can see in the future Dr. George Edwin Hunt, preaching with impassioned face and warning finger upraised, a sermon from this text, *a la* Riley:

"And you young practitioners, you musn't try and hide,
'Cause they's two black supply men astandin' by your side.
And they'll snatch you through the ceilin' 'fore you know what you're about.
Oh! The depot men'll get you
Ef
You
Don't
Watch
Out."

